# Acupuncture Treatment of Guillain–Barré Syndrome After Using Immune Checkpoint Inhibitors: A Case Report

**DOI:** 10.3389/fneur.2022.908282

**Published:** 2022-06-02

**Authors:** Jialing Li, Danghan Xu, Yingyu Liu, Yang Cao, Jun He, Muxi Liao

**Affiliations:** ^1^The First Clinical School of Medicine, The First Affiliated Hospital of Guangzhou University of Chinese Medicine, Guangzhou University of Chinese Medicine, Guangzhou, China; ^2^The First Affiliated Hospital of Guangzhou University of Chinese Medicine, Guangzhou, China; ^3^Guangdong Provincial Key Laboratory of Traditional Chinese Medicine and Acupuncture, Guangzhou, China; ^4^Acupuncture and Rehabilitation Clinical School of Medicine, Guangzhou University of Chinese Medicine, Guangzhou, China

**Keywords:** acupuncture, Guillain–Barré syndrome, immune checkpoint inhibitors, immune-related adverse events, case report, limb weakness, numbness

## Abstract

Guillain–Barré syndrome(GBS) is an autoimmune-mediated peripheral neuropathy. Immune checkpoint inhibitors (ICIs) are the standard treatment for cancer and may lead to immune-related adverse events (irAEs) such as GBS. Corticosteroids, plasma exchange (PE), and intravenous immunoglobulin (IVIG) are currently accepted treatments for ICI-induced GBS. However, there are still adverse reactions, and the effect of relieving symptoms is not as good as expected. Safe and effective complementary replacement therapy to alleviate GBS symptoms and ameliorate the quality of life is urgently required. In this case, a 63-year-old man received ICI therapy and antitumor chemotherapy for lung malignancy. After two courses of treatment, the patient gradually developed limb weakness, numbness, and pain at the ends of the limbs, with cerebrospinal fluid (CSF) albuminocytological dissociation, and electromyography (EMG) suggested demyelinating changes and was diagnosed as GBS. Although the patient received high doses of intravenous gamma globulin and limb weakness symptoms were alleviated, there was still significant numbness and pain in the extremities. After four times of acupuncture treatments, the patient complained that the symptoms of limb numbness and fatigue were significantly alleviated without any discomfort. This case report may provide a new alternative and complementary therapy for immune checkpoint inhibitor-induced GBS, but more definitive and robust evidence is needed to support its efficacy.

## Introduction

Guillain–Barré syndrome (GBS) is an autoimmune-mediated peripheral neuropathy (1). The classic clinical presentation of GBS is symmetrical progressive limb weakness with diminished or absent tendon reflexes (2), and limb paresthesias are also present in most patients. Electromyography (ECG) can detect polyradiculoneuropathy, and cerebrospinal fluid analysis can demonstrate albumin cytology dissociation (3). The incidence of GBS ranges widely (4, 5), with an estimated 100,000 patients diagnosed worldwide each year (1). Otherwise, the incidence rate of GBS increased with age, and the probability of males onset was higher than that of females (3). Symptom severity in GBS typically peaks within 1 month (3), followed by a recovery period of months or years. However, about 5% of patients eventually die due to complications such as respiratory failure, infection, hypotension, and severe cardiac arrhythmias, and about 20% are unable to walk independently (3).

Immune checkpoint inhibitors (ICIs) are widely used in cancer, bringing new hope to patients with advanced cancer. However, ICIs activate the autoimmune system, leading to the attack on normal tissues or organs, resulting in various adverse effects (6). Such adverse reactions caused by ICIs are called immune-related adverse events (irAEs), especially neurological immune-related adverse events, which are rare but potentially life-threatening and require prompt diagnosis and intervention (7). The reported incidence of GBS associated with ICIs is 0.3% (8), and its clinical presentation is not different from classic GBS (9). Plasma exchange (PE) and intravenous immunoglobulin (IVIG) are recognized as methods that can promote rehabilitation and improve disease outcomes in GBS (3). In addition, corticosteroids are the mainstay of treatment for GBS associated with ICIs (10). However, they also cause certain adverse reactions, such as fatigue, pain, anxiety, and so on (11, 12). The effect of alleviating symptoms is not satisfactory, resulting in the impact of the curative effect or quality of life of patients with cancer. Therefore, safe and effective complementary replacement therapy to alleviate the symptoms and enhance the quality of life is urgently required.

Acupuncture is a traditional Chinese medicine therapy. Modern research shows that acupuncture has an excellent curative effect on pain, anxiety, limb dysfunction, and paresthesia and can effectively improve neurological function (13, 14). It has been widely used as a supplementary treatment for cancer pain and neurovascular diseases (15). In this study, we tried acupuncture for a patient with GBS following an ICIs. We surprisingly found that acupuncture completely relieved the patient's symptoms of limb weakness and paresthesia, and the living quality of the patient was enhanced. Here, we report the case in detail.

## Case Report

### Clinical History

A 63-year-old man was admitted to the intensive care unit (ICU) due to coughing up bloody sputum for more than 2 months without apparent cause. Symptomatic treatment was given for anti-infection, hemostasis, phlegm reduction, and nutritional support. After the condition was stable, the patient underwent an ultrasound-guided percutaneous lung biopsy, and the pathology report suggested mucinous adenocarcinoma. The patient's final diagnosis was lung malignancy (mucinous adenocarcinoma cT3N0M1a stage IV). Subsequently, the patient came to our hospital for a 3-week course of antitumor chemotherapy and immune checkpoint inhibitor therapy. The specific regimen was as follows: docetaxel 120 mg and tislelizumab 200 mg. After two courses of treatment, the patient came to our hospital for further treatment. On admission, the patient complained of occasional cough, coughing up a small amount of white sputum and wheezing easily after activities. Symptoms of limb weakness, numbness, and pain in the extremities began to appear 1 week ago, and the patient emphasized that the numbness felt like wearing gloves and socks.

### Clinical and Laboratory Examinations

The size of the bilateral pupils is equal, light reflexes of both pupils were sensitive, and the corneal reflexes were present. The muscle tone of the limbs decreased, the muscle strength of the proximal end of both upper limbs was grade 4, the distal end was grade 2, and the lower limbs were grade 3. The tendon reflexes disappeared symmetrically, and the signs of pyramidal tract and meningeal irritation were negative. Analysis of cerebrospinal fluid showed that the Pandy test was positive, white blood cell (WBC) count was 0, and micro-amount of proteins (M-TP) was 911 mg/L (normal reference value range was 150–450 mg/L), suggesting significant CSF albuminocytological dissociation (Table 1).

**Table 1:** Analysis of cerebrospinal fluid.

**Item**	**Result**		**Unit**	**Reference interval**
Color	Colorless			
Transparency	Clear			
Clot	No clots			
Pandy test	+	*		
RBC	300		E+6/L	
WBC	0		E+6/L	
K	2.91		mmol/L	
Na	145.9		mmol/L	
Cl	125.5		mmol/L	120–132
Glu	3.9		mmol/L	2.50–4.50
M-TP	911	↑	mg/L	150–450
LDH	24		U/L	
ADA	0		U/L	0–8

*RBC, red blood cell; WBC, white blood cell; Glu, glucose level; M-TP, micro-amount of proteins; LDH, lactate dehydrogenase; ADA, adenosine deaminase*.

* *,means different from normal - ; ↑, means higher than normal range*.

### EMG and Neuroimaging

Electromyography showed that bilateral tibial nerves, bilateral common peroneal nerves, bilateral superficial peroneal nerves, bilateral sural nerves, bilateral median nerves, bilateral ulnar nerves, L4-S1, and C5-T1 nerve roots were all damaged. Nerve conduction velocity is slowed, and F-wave latency is prolonged, suggesting nerve demyelination. Brain MRI examination showed mild white matter degeneration, and the rest showed no evident abnormality. Spinal MRI examination showed mild bulging of C5/6, C6/7 intervertebral disks and bulging of L2/3-L5/S1 intervertebral disks. There was no evident stenosis of the spinal canal, and the spinal cord and nerve roots were not compressed. According to the history, symptoms, signs, and auxiliary examinations, we considered the diagnosis of Guillain–Barre syndrome induced by ICIs.

### Acupuncture Treatment

**Figure 1:**
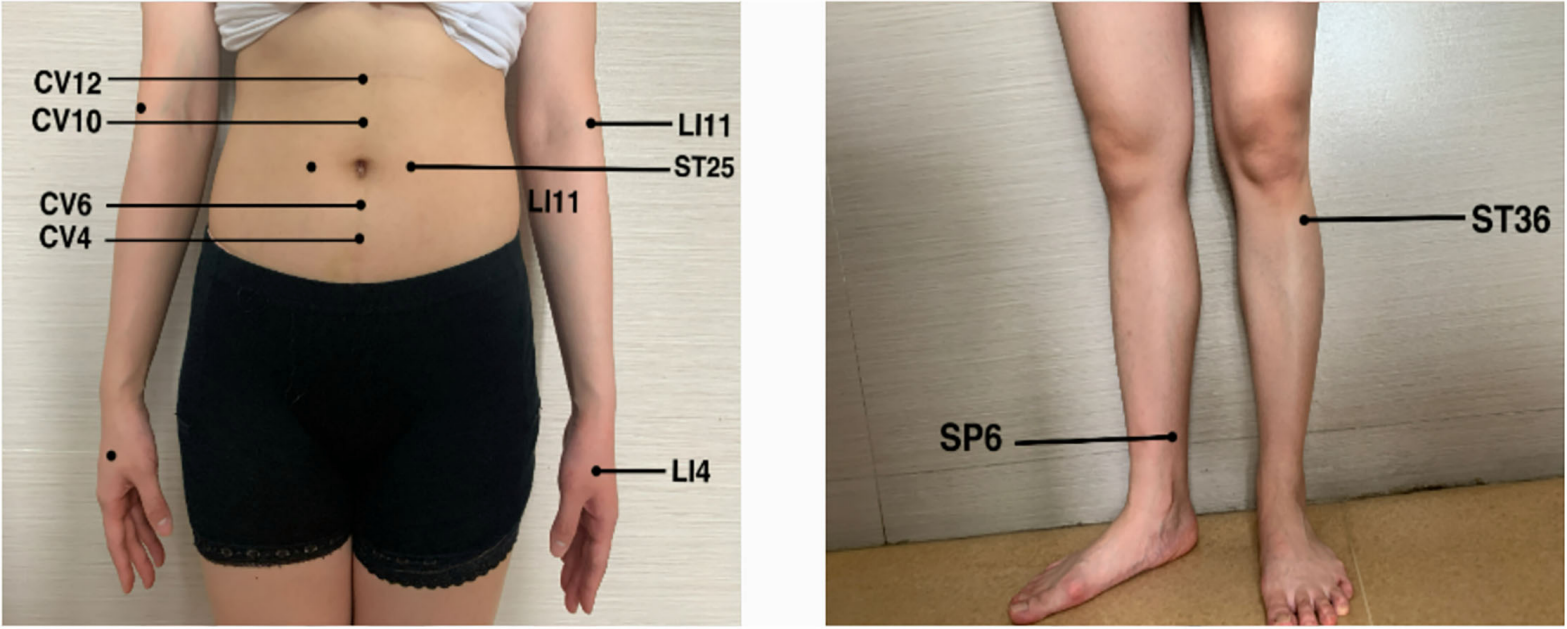
The selected acupoints bilateral Tianshu (ST25), Zhongwan (CV12), Xiawan (CV10), Qihai (CV6), Guanyuan (CV4), bilateral Quchi (LI11), bilateral Hegu (LI4), bilateral Zusanli (ST36) and bilateral Sanyinjiao (SP6).

In the beginning, the patient received a five-day pulse of intravenous gamma globulin, combined with mecobalamin and pregabalin to nourish the nerves. After the end of intravenous immune globulin pulse therapy, the patient complained that the symptoms of limb weakness improved, but there was still noticeable numbness and pain in the extremities. Physical examination showed that the distal muscle strength of both upper limbs increased to grade 3, but there was no significant change in the muscle strength of other limbs. Therefore, an acupuncture doctor with 7 years of experience in acupuncture was arranged to perform acupuncture treatment for him to relieve the weakness and numbness of the limbs and pain. After the skin was disinfected, bilateral Tianshu (ST25), Zhongwan (CV12), Xiawan (CV10), Qihai (CV6), Guanyuan (CV4), bilateral Quchi (LI11), bilateral Hegu (LI4), bilateral Zusanli (ST36), and bilateral Sanyinjiao (SP6) were pierced with stainless steel needles (0.25 mm × 40 mm, TianXie, China). The acupuncturist did not do any manipulations on the acupuncture needles and kept them for 25 min (Figure 1).

**Figure 2:**
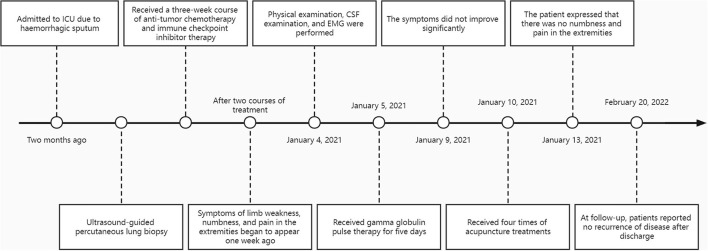
Timeline with relevant data from the episode of care.

After accomplishing the first acupuncture treatment, the patient complained that the symptoms of limb weakness were significantly improved, and the physical examination showed that the muscle strength of the limbs was all grade 4. After completing the second acupuncture treatment, the patient said that there was no limb weakness, and the muscle strength of the limbs could reach grade 5, but numbness and pain at the ends of the limbs were still left. The patient reported that the numbness and pain in the extremities were significantly relieved after finishing the third acupuncture. After four acupuncture treatments, the patient expressed that there was no numbness and pain in the extremities, and there was no adverse acupuncture reaction, which made him extremely satisfied. During the follow-up, the patient stated that the disease had not recurred for 1 year after discharge (Figure 2).

## Discussion

As the most common and most severe autoimmune-mediated acute paralytic neuropathy in neurology (3), GBS is divided into several subtypes, including acute inflammatory demyelinating polyradiculoneuropathy (AIDP), acute motor axonal neuropathy (AMAN), acute motor-sensory axonal neuropathy (AMSAN), Miller Fisher syndrome (MFS), acute panautonomic neuropathy (APN), and acute sensory neuropathy (ASN), the most common subphenotypes being AIDP and AMAN (16). This case can be diagnosed as acute inflammatory demyelinating polyradiculoneuropathy (AIDP) based on the patient's medical history, symptoms, signs, and auxiliary examinations. In addition, since the course of GBS is self-limiting and we did not review CSF analysis before discharge, we need to distinguish it from acute-onset chronic inflammatory demyelinating polyneuropathy (A-CIDP) (17) to prevent doubts about the efficacy of acupuncture in alleviating symptoms. Studies have shown that A-CIDP can only be diagnosed when a patient has GBS and has relapsed or relapsed more than three times 8 weeks after the onset of GBS (2, 17). However, we followed up with this patient, and the patient reported that there had been no recurrence of GBS within 1 year from discharge to the present. Therefore, we can rule out the diagnosis of A-CIDP.

It is worth noting that antitumor chemotherapy drugs can also induce peripheral neuropathy, such as taxanes, platinums, vinblastines, and so on (18), which we call chemotherapy-induced peripheral neuropathy (CIPN) (19). CIPN is a neuropathy mainly involving sensory nerves (20). It is characterized by symmetrical numbness, paresthesia, pain, and hyperesthesia at the distal end of the limb, with a typical “glove”–“sock” distribution (21), which is almost the same as the sensory symptoms presented by GBS. In this case, the patient also used docetaxel for antitumor chemotherapy while using ICIs. Docetaxel belongs to the taxane class of antitumor drugs and has the risk of inducing CIPN. Therefore, we do not rule out the possibility of additional adverse reactions of docetaxel for the symptoms of numbness and pain in the patient's extremities.

The exact etiology of classic GBS is unknown, researchers consider that the infection of the peripheral nerves or nerve roots leads to extensive inflammatory demyelinating lesions in the peripheral nervous system (22). Most patients had a history of infections, mainly upper respiratory tract infections and gastrointestinal infections, within the 4 weeks before the onset of neurological symptoms (23). In addition, the pathogenesis of GBS is also related to the factors such as vaccination (24), ganglioside administration (25, 26), and surgery (27). Studies have shown that 50–70% of cases occur 7–14 days after infection or immune stimulation, which would induce an abnormal autoimmune response to the peripheral nerve and its spinal nerve roots (28–30). The molecular mimicry between pathogen antigens and neural antigens is currently considered to be one of the most important mechanisms leading to the pathogenesis of GBS (3). This theory holds that some components of pathogens have the same structure as some components of peripheral nerves, and the body's immune system recognizes them incorrectly. Autoimmune cells and autoantibodies conduct immune attacks on normal peripheral nerves, resulting in peripheral nerve demyelination. Studies have currently supported the vital role of molecular simulation in GBS pathogenesis (31). As a type of immune stimulation, immune checkpoint inhibitors may also induce abnormal autoimmune responses in the body, resulting in peripheral nerve demyelination.

Immune checkpoint inhibitor-induced GBS usually develops after three courses of ICIs, and the disease progresses rapidly (2). CSF analysis showed albuminocytological dissociation, and electrophysiological studies supported demyelinating neuropathy (32). The mechanism of ICI-induced GBS is currently unclear. It may be due to the abolition of self-tolerance that activates cytotoxic T lymphocytes while reducing the inhibition of antibody-producing B lymphocytes (2). It is worth noting that the treatment of ICI-induced GBS is different from that of classic GBS. Although corticosteroids are not recommended for classic GBS, they can significantly improve clinical symptoms of ICI-induced GBS (33).

Through reviewing the previous literature on GBS related to ICIs, we found that the majority of patients (44%) had a 73% improvement in clinical symptoms when receiving IVIg and steroids concurrently. In contrast, the clinical efficacy of PE is uncertain due to the small number of effective cases (33). The American Society of Clinical Oncology (ASCO) clinical practice guidelines state that in the treatment of GBS associated with ICIs, IVIG 0.4 g/kg/day should be used for 5 days in combination with (methyl)prednisolone 1–2 mg/kg/day (34). The National Comprehensive Cancer Network (NCCN) guidelines recommend high-dose intravenous methylprednisolone 1 g/d for 5 days with concurrent IVIG and PE (35). However, the European Society for Medical Oncology (ESMO) considers that 1–2 mg/kg/day of (methyl)prednisolone is sufficient for general ICIs-related GBS. IVIG and PE are recommended when symptoms do not improve or worsen (36). According to the guideline recommendations and previous literature, once ICIs-related GBS is diagnosed, immunotherapy should be stopped immediately. First-line treatment is recommended to use IVIg 0.4 g/kg/day for 5 days, and concurrently use (methyl)prednisolone 1–2 mg /kg/day. Second-line treatment is PE, which should be considered when symptoms do not improve or when the condition worsens (33).

In this case, the patient only received IVIG therapy and was not assigned corticosteroid therapy, which could not effectively relieve the clinical symptoms caused by ICI-related GBS. After four times of acupuncture treatments, the patient's symptoms of limb weakness and numbness and pain were successfully relieved, the quality of life was improved, and there were no adverse reactions. The patient expressed that he was satisfied with the curative effect of acupuncture.

As the primary treatment modality of East Asian medicine, acupuncture and moxibustion have received extensive attention worldwide (37). In the clinical application of acupuncture, the functional connection and communication between acupuncture points, the brain (the heart of ancient medicine), and the gut are essential (38), similar to the gut–brain axis in modern medicine. The gut–brain axis represents bidirectional communication between the gut and the brain (39, 40) and plays an important role in neurodegenerative diseases. Dysbiosis of the gut microbiota will result in an imbalance of the gut–brain axis, which will induce an increase in inflammatory signaling factors and epithelial permeability (38). The vagus nerve has been reported to be important in gut microbiota–brain axis communication, and acupuncture may exert immunomodulatory effects through the vagus nerve-mediated regulation of the brain–gut axis (41), further exerting neuroprotective effects (42).

In this case, the acupuncturist selected Tianshu (ST25), Zhongwan (CV12), Xiawan (CV10), Qihai (CV6), Guanyuan (CV4) as the local abdominal acupoints, Zusanli (ST36), Sanyinjiao (SP6), Quchi (LI11), and Hegu (LI4) were selected as the distal points of the limbs. Studies have shown that acupuncture at Zusanli (ST36), Sanyinjiao (SP6), and Tianshu (ST25) can regulate the gut–brain axis and treat diseases with gut–brain interaction disorders (43). Studies have confirmed that acupuncture at Zusanli (ST36) can enhance immunity and improve exercise capacity, which may be related to the modulation of gut microbial dysbiosis, thereby inhibiting neuroinflammation (38). Zhongwan (CV12), Xiawan (CV10), Qihai (CV6), and Guanyuan (CV4) are all on the route of the conception vessel. The digestive tract is a tubular object located on the midline of the human body in the early stage of embryogenesis, which is the route of the conception vessel. We believe that acupuncture at these acupoints may regulate the gut–brain axis by stimulating the conception vessel to achieve immune regulation and neuroprotection effects. In addition, previous studies have shown that acupuncture at Quchi (LI11), Hegu (LI4), Zusanli (ST36), and Sanyinjiao (SP6) can alleviate the symptoms of limb numbness effectively (44). However, the specific mechanism is not precise. It may be due to the vital nerve distribution where these acupoints are located, and stimulating them can effectively alleviate numbness, pain, and other paresthesias caused by neuropathy. Still, we need further research to clarify it.

It should be noted that the patient also used mecobalamin and pregabalin while using IVIG to nourish the nerves and improve the symptoms of limb weakness, numbness, and pain. At this point, the effect of acupuncture on relieving symptoms may be questioned. Relevant studies have pointed out that acupuncture has synergistic and attenuating impact in managing cancer-related symptoms and adverse reactions of anticancer therapy (45, 46). In this case, the effects of acupuncture, mecobalamin, and pregabalin are synergistic, and acupuncture can increase their efficacy in nourishing nerves and improving symptoms, which provides a reference for our clinical treatment. In the process of routine use of western medicine, acupuncture can be considered to make the onset time of the medicine faster and the effect better.

According to the latest published reviews and clinical studies, there is no literature to collect and evaluate the clinical evidence of acupuncture for GBS (47). There is only one related article in the English literature, which is a clinical case of acupuncture for the treatment of CIDP (48). Most of the cases and clinical studies of acupuncture in the treatment of GBS are published in Chinese literature. The sample size of these related clinical studies is relatively small, the research center is single, and the quality of the research is low. Therefore, from the perspective of evidence-based medicine, if we want to obtain clinical evidence that acupuncture effectively treats GBS, it is necessary to carry out large-sample, multi-center, high-quality clinical research and basic research.

## Conclusion

In conclusion, our case shows that acupuncture eliminated the symptoms of weakness and numbness and pain in ICI-related GBS patient's limbs and improved their quality of life. Recent research shows that acupuncture has advantages in treating neurological disorders (49). Based on our experience, we believe that acupuncture may be an effective, economical, and safe complementary therapy for treating GBS. However, we still need high-quality, large-sample clinical research and basic research to demonstrate the mechanism of action and efficacy of acupuncture for GBS.

## Data Availability Statement

The original contributions presented in the study are included in the article/Supplementary Material, further inquiries can be directed to the corresponding author/s.

## Ethics Statement

The studies involving human participants were reviewed and approved by Ethics Committee of the First Affiliated Hospital of Guangzhou University of Traditional Chinese Medicine and obtained ethical approval (Ethics Batch Number: K-2022-018). The patients/participants provided their written informed consent to participate in this study. Written informed consent was obtained from the individual(s) for the publication of any potentially identifiable images or data included in this article.

## Author Contributions

JL and DX conceived the idea, conceptualized the research, and prepared the manuscript. YC and YL collected and analyzed the data. JH and ML reviewed by Manuscript. All authors contributed to the article and approved the submitted version.

## Funding

This study was supported by Guangdong Provincial Key Laboratory of Traditional Chinese Medicine and Acupuncture (06), Zhangjiawei National Famous and Old TCM Experts Inheritance Studio (National Administration of Traditional Chinese Medicine [2012] No. 149), the special fund Innovating and Strengthening the Hospital for preventive treatment of disease of the First Affiliated Hospital of Guangzhou University of Chinese Medicine (2019ZWB07), and Guangdong Province Rural Science and Technology Commissioner Project (KTPYJ2021026).

## Conflict of Interest

The authors declare that the research was conducted in the absence of any commercial or financial relationships that could be construed as a potential conflict of interest.

## Publisher's Note

All claims expressed in this article are solely those of the authors and do not necessarily represent those of their affiliated organizations, or those of the publisher, the editors and the reviewers. Any product that may be evaluated in this article, or claim that may be made by its manufacturer, is not guaranteed or endorsed by the publisher.
