# Supplementary material for: Acupuncture Treatment of Guillain–Barré Syndrome After Using Immune Checkpoint Inhibitors: A Case Report
Source: Front Neurol. 2022 Jun 2;13:908282. doi: 10.3389/fneur.2022.908282 (PMC9201402; doi:10.3389/fneur.2022.908282)
Supplement: Supplementary file 1 [file Data_Sheet_1.pdf]

# Electromyogram report

**Motor nerve conduction velocity measurement**

| Nerve | Latency period (ms) | Amplitude (mV) | Distance (mm) | Latency difference (ms) | Segments | Speed (m/s) |
|---|---|---|---|---|---|---|
| Tibial.L | | | | | | |
| Ankle | 7.5 | 1.9 ↓ | | 12.4 | Abductor hallucis-Ankle | |
| Popliteal fossa | 19.6 | 1.6 ↓ | 360 | 13.2 | Ankle-Popliteal fossa | 30 ↓ |
| Tibial.R | | | | | | |
| Ankle | 7.5 | 1.9 ↓ | | 12.3 | Abductor hallucis-Ankle | |
| Popliteal fossa | 20.4 | 1.6 ↓ | 360 | 10.6 | Ankle-Popliteal fossa | 28 ↓ |
| Peroneal.L | | | | | | |
| Ankle | 6.6 | 0.1 ↓ | | 5.9 | Extensor digitorum brevis-Ankle | |
| Fibula (head) | 20.0 | 0.1 ↓ | 300 | 11.7 | Ankle-Fibula (head) | 22 ↓ |
| Popliteal fossa | 23.0 | 0.1 ↓ | 70 | 12.7 | Fibula (head)-Popliteal fossa | 23 ↓ |
| Peroneal.R | | | | | | |
| Ankle | 12.6 | 0.9 ↓ | | 14.9 | Extensor digitorum brevis-Ankle | |
| Fibula (head) | 33.7 | 0.3 ↓ | 300 | 12.1 | Ankle-Fibula (head) | 14 ↓ |
| Popliteal fossa | 37.6 | 0.4 ↓ | 70 | 9.2 | Fibula (head)-Popliteal fossa | 18 ↓ |
| Median.L | | | | | | |
| Wrist | 8.8 | 0.7 ↓ | | 9.8 | | |
| Elbow | 15.7 | 0.5 ↓ | 200 | 6.3 | Wrist-Elbow | 29 ↓ |
| Median.R | | | | | | |
| Wrist | 7.6 | 0.7 ↓ | | 9.3 | | |
| Elbow | 15.7 | 0.8 ↓ | 220 | 8.4 | Wrist-Elbow | 27 ↓ |
| Ulnar.L | | | | | | |

| | | | | | | |
|---|---|---|---|---|---|---|
| Wrist | 4.3 | 2.1 | | 11.1 | Abductor digiti minimi (manus)-Wrist | |
| Below elbow | 12.2 | 1.1 | 200 | 7.7 | Wrist-Below elbow | 25 ↓ |

Ulnar.R

| | | | | | | |
|---|---|---|---|---|---|---|
| Wrist | 3.7 | 2.0 ↓ | | 10.9 | Abductor digiti minimi (manus)-Wrist | |
| Below elbow | 12.2 | 1.3 ↓ | 200 | 7.8 | Wrist-Below elbow | 24 ↓ |

**F-Wave**

| Nerve | M-wave latency | F-wave latency | F-wave occurrence rate (%) |
|---|---|---|---|
| Median.L | 10.8 | 32.5 ↑ | 100.0 |
| Median.R | 11.6 | 33.6 ↑ | 96.7 |
| Ulnar.L | 7.9 | Cannot be elicited | |
| Ulnar.R | 6.6 | Cannot be elicited | |

**Sensory nerve conduction velocity measurement**

| Nerve | Latency period (ms) | Amplitude (mV) | Segments | Distance (mm) | Speed (m/s) |
|---|---|---|---|---|---|
| Superficial peroneal.L | | | | | |
| Ankle | 2.6 | 3.0 ↓ | Dorsum of foot-Ankle | 70 | 27 ↓ |
| Superficial peroneal.R | | | | | |
| Ankle | 2.4 | 2.8 ↓ | Dorsum of foot-Ankle | 70 | 29 ↓ |
| Sural.L | | | | | |
| Lower leg | 5.3 | 3.1 ↓ | Ankle-Lower leg | 150 | 28 ↓ |
| Sural.R | | | | | |
| Lower leg | 5.2 | 3.6 ↓ | Ankle-Lower leg | 150 | 29 ↓ |
| Median.L | | | | | |
| Wrist | 4.4 | 4.6 ↓ | Digit Ⅲ(long finger)-Wrist | 130 | 30 ↓ |
| Median.R | | | | | |
| Wrist | 4.8 | 4.5 ↓ | Digit Ⅲ(long finger)-Wrist | 130 | 27 ↓ |
| Ulnar.L | | | | | |
| Wrist | 3.7 | 2.9 ↓ | Digit Ⅴ(little finger)-Wrist | 100 | 27 ↓ |
| Ulnar.R | | | | | |
| Wrist | 3.6 | 2.1 ↓ | Digit Ⅴ(little finger)-Wrist | 100 | 28 ↓ |

**H reflex**

| Nerve | Latency period (ms) | Amplitude(max) (mV) |
|---|---|---|
| Tibial.L | | |
| M-Wave | 7.0 | 1.9 |
| H-Wave | Cannot be elicited | |
| Tibial.R | | |
| M-Wave | 7.7 | 1.3 |
| H-Wave | Cannot be elicited | |
